# Consensus‐Based Guidelines for Communicating a Misdiagnosis of Multiple Sclerosis to Reduce Psychological Distress

**DOI:** 10.1002/brb3.70109

**Published:** 2024-10-28

**Authors:** Angela Lesley Baufeldt, Nikos Evangelou, Nima Moghaddam, Mark Gresswell, Roshan das Nair

**Affiliations:** ^1^ School of Psychology University of Lincoln Lincoln UK; ^2^ Mental Health and Clinical Neurosciences, School of Medicine University of Nottingham Nottingham UK; ^3^ Health Division SINTEF Trondheim Norway

**Keywords:** communication, Delphi, guidelines, misdiagnosis, multiple sclerosis

## Abstract

**Background:**

Multiple sclerosis (MS) misdiagnosis is common, and when discovered, frequently leads to substantial disruption to patients’ lives and anxiety for clinicians. Our objective was to develop expert consensus‐based guidelines about how to communicate a misdiagnosis of MS to a patient, to reduce the potential for both psychological distress and litigation.

**Methods:**

A modified Delphi method using a systematic literature review on doctor and patient experiences of the MS diagnosis communication was used to populate items for a first‐round questionnaire. Our Delphi panel represented three perspectives (clinicians, people with MS, and published experts in health communication), and we recruited 18 panelists in total (6 per perspective). Consensus was defined a priori as 75% of panelists giving an item the same rating. A feedback round was undertaken with six external reviewers, naïve to the guideline development process, and the panelists. Items were reviewed by the study team and synthesized to create the finalized guidelines.

**Results:**

Consensus was reached for 45 items rated as “very important” and presented in the feedback round. The study team synthesized the 45 items to 27 items. Ten items related specifically to the communication of the MS misdiagnosis and 17 items to generic guidelines highlighted as important in the MS misdiagnosis appointment. Seven recommendations form the guidelines presented here.

**Conclusions:**

Seven consensus‐based recommendations offer guidance to practising neurologists in their communication with patients in a situation that has the potential to be highly distressing, for both clinician and patient.

## Graphical abstract

This consensus study used a modified Delphi technique to identify very important items to be considered for inclusion in clinical guidelines for communicating a misdiagnosis of multiple sclerosis (MS). Items identified as being specific to the MS misdiagnosis communication were embedded with psychological principles known to contribute to the therapeutic relationship between doctor and patient. The outcomes of this systematic process are seven recommendations for doctors to use when communicating a misdiagnosis to a patient in a psychologically‐informed way that aims to reduce the distress for the patient and the doctor.

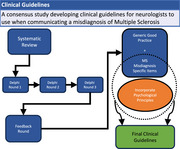

## 1 Introduction

Globally, an estimated 2.8 million people reported living with multiple sclerosis (MS) in 2020, of which almost one million were from the USA (The Multiple Sclerosis International Federation 2020). Despite the availability of diagnostic guidelines (Thompson et al. 2018), the lack of a single, diagnostic biomarker in MS increases the likelihood of misdiagnosis, an incorrect assignment of a diagnosis. MS misdiagnosis is attributed to various factors, including neurologists’ overreliance on imaging (Solomon and Weinshenker 2013), misapplication of diagnostic criteria (Solomon et al. 2021), and not excluding conditions that mimic MS (Calabrese et al. 2019.). Prevalence rates of MS misdiagnosis have been reported to be as high as 18%–31% (Kaisey et al. 2019; Yamout et al. 2017). The most common alternative diagnoses found for those initially misdiagnosed with MS were migraine (Kaisey et al. 2019), psychogenic (Yamout et al. 2017) and vascular disease (Nicolau, de Oliveira, and Bichuetti 2015). The consequences of carrying a misdiagnosis can be significant for a patient, such as unnecessary exposure to disease‐modifying treatments (DMTs), some with significant side effects (Kaisey et al. 2019); and the actual condition not receiving appropriate treatment (Solomon and Weinshenker 2013) leading to increased morbidity (Solomon et al. 2016). Incorrect diagnosis can lead to patients making unnecessary or inappropriate life‐altering decisions, such as choosing not to have children (Alwan et al. 2013), to change career (Bogosian et al. 2009), or to take early retirement (Bogenschutz et al. 2016). Therefore, the removal of an MS diagnosis can be at times more significant than receiving the diagnosis in the first place (Crouch 2016). Many neurologists consider the prospect of telling a patient they have been misdiagnosed as more daunting than communicating a diagnosis of MS, with some choosing not to (Solomon, Klein, and Bourdette 2012). Fear of causing distress is one of the most common reasons for not communicating a misdiagnosis to a patient (Solomon, Klein, and Bourdette 2012; Anestis et al. 2021). In addition, litigation, with the potential to affect a clinician's career, is a significant barrier to effective communication (Wu et al. 1997).

The manner in which a doctor communicates information to patients is known to impact on the patient's relationship with healthcare professionals, services, and treatment compliance (Tuckett 2004). Patients who report their MS diagnosis as a positive experience are more likely to be open about their diagnosis with friends, family, and colleagues, and have better psychosocial outcomes than those who reported having a negative experience (Leavitt et al. 2022). There are economic implications of poor healthcare communication; for example, patients who were unsatisfied with the explanation of a functional neurological disorder diagnosis had substantially higher healthcare costs than those who reported the experience as satisfactory (Walzl, Solomon, and Stone 2022).

Numerous research reports on poor patient satisfaction with MS diagnostic communication with common issues, including appointments being too short (Kaufmann et al. 2018); content not tailored to their situation (Messina et al. 2015); an unclear diagnosis (Kamm et al. 2020); patients not adequately emotionally supported (Messina et al. 2015); and being informed of their diagnosis via telephone or letter (Messina et al. 2015). Doctors’ experiences of communicating the MS diagnosis included feelings of moderate to high anxiety (Anestis et al. 2021); an avoidance of saying “MS,” preferring descriptive terms (Heesen et al. 2003) or only saying “MS” at the end of the appointment, or at a follow‐up appointment (Solari et al. 2007), or after monitoring the patient's emotional reaction (Martinelli et al. 2012); and finding time‐limited appointments a challenge when attempting to convey all necessary information (Price, Lucas, and Lane 2021). Patients are therefore unlikely to experience the communication of their diagnosis in a satisfactory way, and clinicians are faced with many barriers to providing effective communication (Anestis et al. 2021).

Recommendations on how to communicate an MS misdiagnosis are available, although this is limited (Coebergh, Wren, and Mumford 2014; Boissy and Ford 2012). Articles identified acknowledge the challenge of communicating a misdiagnosis of MS, and the risk to the doctor–patient relationship. Coebergh, Wren, and Mumford (2014) share their experiences of patients’ strong negative emotional reactions of having their MS diagnosis removed and present suggestions of how to have this conversation.

Although communicating an MS *diagnosis* is routine for neurologists, some patients report poor satisfaction with this interaction. If patients’ experiences of the *diagnostic* communication are variable, and clinicians report communicating a misdiagnosis to be more challenging than a diagnosis, then there is a clear and urgent need to support clinicians in communicating a misdiagnosis effectively. Therefore, the objective of this study was to develop consensus‐based clinical guidelines that a neurologist can use to reduce the potential for psychological distress when communicating a misdiagnosis of MS.

## 2 Methods

The Delphi technique is a consensus method that uses an iterative survey format with controlled feedback to a group of informed experts in order to gain consensus on a particular issue (Keeney, Hasson, and McKenna 2011).

### 2.1 Panelist Recruitment

Three equal subgroups of experts were purposively chosen for their expertise and experience and included clinicians, experts‐by‐experience (EBE), and published experts. These three groups were chosen to reflect both the clinician and patient perspectives, as well as those with expertise in healthcare communication. Inclusion criteria required potential participants to be 18 years or older, could communicate in English, had access to a device with internet connection, and identified as belonging to one of the three subgroups. Clinicians were professionals directly involved in patient care for those diagnosed with MS (*n* = 6). EBE were people who had a diagnosis of MS (*n* = 6). Published experts were people with expertise in communication or who disseminated content related to MS or medical communication (*n* = 6). The panelists’ expertise was verified by the guideline development group (the authors of this article). Panelists could self‐identify as belonging to more than one expert group, for example, be an EBE and a published expert. No geographical limitations were imposed. Consistent with the Delphi method, anonymity between panelists was maintained throughout.

Participants who were interested but could not participate in the Delphi rounds (due to over‐recruitment) were invited to be external reviewers in a feedback round to comment on items that gained consensus.

### 2.2 Standard Protocol Approvals, Registrations, and Patient Consent

This study was given a favorable opinion by the University of Lincoln Research Ethics Committee; ethics reference UoL2021_7161. All panelists and external reviewers gave informed consent to participate in this research.

### 2.3 Round 1 Questionnaire

An initial review of empirical research investigating how neurologists communicate a misdiagnosis of MS to patients yielded insufficient results. A systematic literature review of doctors’ and patients’ perspectives on communicating an MS diagnosis was conducted instead. PsycINFO, CINAHL, MEDLINE, Scopus, and Google Scholar incognito were searched in August 2021 for eligible articles (see the Appendix for search strategy). The PRISMA 2020 statement (Page et al. 2021) was followed, and a review protocol was prospectively registered with PROSPERO (CRD42021273697). The study team extracted items from this review and supplemented these with items from guidelines in cancer (Baile et al. 2000), dementia (Peixoto, Diniz, and de Oliveira Godeiro 2020), and breaking bad news (Rabow and Mcphee 1999; Narayanan, Bista, and Koshy 2010; Vandekieft 2001). Guidelines from these areas were used in this study as they were identified during the search but excluded due to not meeting the criteria for the systematic review. Extracted items were principles and practices suggested by the authors of included studies as considerations for diagnostic communication from clinician and patient perspectives. A total of 82 items were identified for inclusion in the questionnaire. Similar principles and practices of the communication were grouped together into five domains, which formed the structure of the questionnaire. These five domains included preparation (20 items); delivery of information (20 items); managing emotions (12 items), checking understanding, and clarifying (8 items); and summary, strategy, and signposting (22 items).

### 2.4 Data Collection

The study team decided, in advance, to have three Delphi rounds because this provides sufficient opportunity for consensus to develop, with additional rounds resulting in higher attrition rates (Keeney, Hasson, and McKenna 2011). Therefore, three questionnaires were developed over the duration of the Delphi study. Panelists had 2 weeks to complete the questionnaire, and the study team had 2 weeks to develop the subsequent questionnaire.

Panelists received a link, via e‐mail, to complete the consent form, demographic form, and Round 1 hosted on the Qualtrics platform. Panelists could rate a proposed item as “very important,” “somewhat important,” “not important,” or they could “actively reject” the item for inclusion into the guidelines. Items that reached consensus after a round were presented to panelists at the beginning of the next round for review and commentary. Items not reaching consensus were analyzed and carried forward to the next round.

A feedback round was incorporated into the study design to ensure applicability of the draft recommendations. During the feedback round, items that gained consensus as “very important” were presented to panelists and external reviewers for their comments. This information was considered by the study team and used to inform the final guidelines.

### 2.5 Data Analysis

Each round elicited qualitative feedback from panelists, for example, suggestions for item amendments, additional items, and justifications for ratings. Qualitative feedback relating to an item was anonymously carried forward to the next round for panelists to use when reconsidering their response. Qualitative content not related to a specific item was discussed amongst the study team for consideration of generating new items or changing the phrasing or wording of an item. Any disagreements were resolved through discussion. Consensus was defined a priori as ≥ 75% of panelists agreeing the same rating for an item (Diamond et al. 2014).

## 3 Results

Demographic data for the panelists and external reviewers are presented in Tables 1 and  2. Two panelists did not contribute past Round 1, a trainee neurologist and a Professor of Communication.

**TABLE 1:** Demographics of the Delphi panelists who completed all three rounds.

	*n* (%)
Panelists	16
Women	10 (63)
Men	6 (33)
Age in years	
Median (range)	42 (34–63)
Ethnicity	
Asian or Asian British	1 (6)
White	15 (95)
Group 1: Clinicians	5
Specialism	
Consultant neurologist	3 (19)
Clinical psychologist	1 (6)
MS nurse specialist	1 (6)
Years in practice	
Median (range)	15.5 (7–31)
Group 2: Experts‐by‐experience	6
Age at diagnosis (in years)	
Median (range)	47 (24–53)
Time since diagnosis (in years)^a^	
Median (range)	10 (7–14)
Received treatment for MS, for example, DMT	
Yes	4
No	1
Missing	1
Group 3: Published experts	5
Book author on MS	1 (6)
Professor of communication	2 (13)
Social media content manager	1 (6)
CEO organization for healthcare communication	1 (6)
Years contributing to the field	
Median (range)	10.5 (3–25)

Abbreviations: DMT, disease‐modifying treatment; MS, multiple sclerosis.

^a^ Missing data for one experts‐by‐experience (EBE).

**TABLE 2:** Demographics of the external reviewers.

	*n* (%)
External reviewers	6
Women	5 (83)
Declined to answer	1 (17)
Age in years	
Median (range)	37 (32–57)
Ethnicity	
White	5 (83)
Declined to answer	1 (17)
Group identified with	
Group 1: Clinician	2
Group 2: Expert‐by‐experience	3
Group 3: Published expert	2

### 3.1 Delphi Results

In Round 1, the questionnaire contained 82 items for panelists to rate. Consensus was reached for 33 items (40%), including one item that was rejected (“the doctor should tell a patient's family before telling the patient”), whereas four items were combined into one item, and five items were reworded.

In Round 2, panelists rated 45 items. Consensus was reached for four items (9%), 18 items were reworded, and two new items were created. An additional question was included in Round 2 regarding adaptions to clinical practice clinicians and services had to make during the Covid‐19 pandemic (see Table 3). Despite the lowest three options reaching consensus, comments by panelists indicated strongly that these options were not appropriate and should not be considered options for communicating this information.

**TABLE 3:** Consensus for adaptions to clinical practice *n* = 16.

The doctor should communicate an MS misdiagnosis:		Consensus (%)
1	In person, during a face‐to‐face appointment	100
2	Over a video call using Microsoft Teams, Zoom, or another virtual platform	94
3	Over a telephone call	88
4	In a letter detailing the misdiagnosis	81
5	In an email detailing the misdiagnosis	81
6	In a text message detailing the misdiagnosis	75

Abbreviation: MS, multiple sclerosis.

In Round 3, the questionnaire contained 43 items for panelists to rate. Consensus was reached for 22 items (54%). Of the items that reached consensus, nine were rated as “somewhat important,” one “not important,” and one was rejected by panelists (“the doctor should postpone telling the patient of the misdiagnosis if the doctor feels the patient is not ready to hear the information”). The full results of the 45 items that reached consensus as “very important” are presented in Table 4.

**TABLE 4:** Delphi items reaching consensus as “very important” items.

Item	Consensus^a^ (%)
**I. Preparation**	
1. The doctor should schedule an appointment with the patient as soon as they are aware the patient has been misdiagnosed	89
2. The doctor should be familiar with the patient's case by reading through their clinical notes prior to the appointment	94
3. The doctor should ensure the space where the appointment takes place allows for privacy, that is, not in a shared space with colleagues working nearby and on a ward	89
4. The doctor should ensure enough time is allocated for the appointment	94
5. The doctor should consider where the appointment takes place and, where possible, limit opportunities for interruptions, for example, placing telephone on silent/do not disturb and not on a ward	94
6. The doctor should use an interpreter if they cannot communicate with the patient in the same language	89
7. The doctor should ensure the space where the appointment takes place has seating available	78
**II. Delivery of the information**	
8. The doctor should take time at the start for the appointment to build rapport with the patient	81
9. The doctor should be unhurried	78
10. The doctor should actively listen to the patient	100
11. The doctor should tailor their information to the unique situation of the patient	83
12. The doctor should tailor their communication to the patient	82
13. The doctor should explore with the patient the patient's understanding of their current medical situation	94
14. The doctor should review the patient's current medical information and any treatments they are currently receiving	89
15. The doctor should be honest and transparent in their interaction with the patient	89
16. The doctor should discuss why the diagnosis of multiple sclerosis is in doubt	100
17. The doctor should provide a summary of the reason (e.g., following on from the tests the patient completed) for the appointment and that this new information changes the understanding of the patient's diagnosis.	88
18. The doctor should use clear and straightforward language.	94
19. The doctor should not use euphemisms, for example, demyelinating condition/inflammatory disease instead of multiple sclerosis	81
20. The doctor should explain the meaning of technical/medical terms to the patient if they need to use these terms in the appointment. The use of analogies may assist the doctor in doing so effectively	75
21. The doctor should deliver information in manageable amounts at a time, pausing and checking patient understanding before asking the patient if they are alright to go on	88
22. The doctor should provide an explanation of why the diagnosis of multiple sclerosis was initially given to the patient, if this information is known	94
23. The doctor should say “we do not know your diagnosis” instead of offering an alternative diagnosis if the diagnosis is unknown	81
**III. Managing emotions**	
24. Once the doctor has communicated the news, they should allow the patient time to process this; for example, sitting in silence for a while, or allowing them to express whatever emotion arises	78
25. The doctor should provide adequate emotional support for the patient. Where they are unable to do this, they should support the patient to access the appropriate services	88
26. The doctor should respond with empathy if/when they observe the patient becoming distressed by asking relevant questions to elicit thoughts and feelings	82
27. The doctor should be prepared to apologize to the patient as most appropriate to the situation. For example, “I'm sorry to be the one to tell you…” or “I can see that this has come as a surprise to you, and I can only imagine how confusing this must be right now. I am sorry that this has not been straightforward for you”	81
28. The doctor should acknowledge the impact of removing the diagnosis from the patient might have (especially if the patient has carried the diagnosis for many years)	89
**IV. Checking understanding and clarifying**	
29. The doctor should ask the patient for their understanding of what they have been told	100
30. The doctor should explore with the patient what this news means to them	82
31. The doctor should check if the patient understands the need to stop treatment, if they are receiving any for multiple sclerosis	88
32. The doctor should encourage the patient to ask questions	94
33. The doctor should check what support the patient has for them, for example, family, friends, and social network. If the person has no support around them the doctor should be able to provide signposting to helplines or other support and resources, the person can access	88
**V. Summary, strategy, and signposting**	
34. The doctor should summarize the main points of the appointment to the patient	82
35. The doctor should summarize why the diagnosis of multiple sclerosis is incorrect for the patient	88
36. The doctor should discuss the patient's treatment options given the change in their diagnosis	88
37. The doctor should clarify if they think a different diagnosis offers a better explanation for the patient's symptoms	94
38. The doctor should let the patient know if there is not enough information to diagnose their symptoms	76
39. The doctor should discuss whether the patient needs further tests/diagnostic investigation	88
40. The doctor should consider discussing the implications of removing a diagnosis of multiple sclerosis at a follow‐up appointment. For example, legal implications of driving and impact on claiming benefits	88
41. The doctor could ask what they can do to help the patient adjust to the change in their diagnosis	81
42. The doctor should let the patient know they will notify, in writing, the patient's general practitioner/primary care physician of the change in diagnosis	82
43. The doctor should offer a follow‐up appointment with whomever is most appropriate for the patient. This may include a follow‐up appointment with the Neurologist, an MS nurse, clinical psychologist, or another relevant professional best suited to meet the patient's needs	100
44. The doctor should provide the patient with information about what happens next for them, for example, discharge and referral to other services	94
45. The doctor should provide tailored information to the patient in written form	81

^a^ Consensus defined as 75% or more panelists rating an item as “very important.”

Areas of disagreement between panelists occurred when considering whether doctors should apologize to a patient regarding the MS misdiagnosis and how this should be done. Despite the panel's reservation regarding an apology, this is, in fact, a statutory requirement in some countries, like the United Kingdom (Care Quality Commission 2022), and examples describing how to apologize are available (General Medical Council; The Nursing, and Midwifery Council 2022). Comments from panelists highlighted the necessity that the apology is genuine and sincere: “Hmm. I'd feel incredibly patronised. I'd think they were mouthing the apology. Empathy is one thing. Fake apology another. Besides, it opens legal door. They [sic] lawyers would faint if they heard that. What if it were recorded?”, whereas another panelist commented “I think this [apologising] is powerful.” A panelist in a later round suggested a reconcilement of this difficulty “Sorry is a powerful word. The Dr [sic] needs to be empowered to do this by the legal team.”

### 3.2 Feedback Round Results

The feedback round contained the 45 items that reached consensus as being “very important.” External reviewers (*n* = 6) and panelists (*n* = 16) reviewed the final 45 items and had the opportunity to provide feedback.

## 4 Final Synthesis of Guidelines

We reviewed the final 45 items and collapsed similar items together (see Table 5 for an example), thereby reducing the number to 27 items. Of the 27 items, 17 can be found in generic guidelines (NICE 2021), and 10 items are specific to the MS misdiagnosis communication (see Table 5).

**TABLE 5:** Example of multiple items condensed into a single item.

Item number	Item	Final item
1	The space where the appointment takes place allows for privacy, that is, not in a shared space with colleagues working nearby and on a ward	The appointment should take place in an appropriate setting that provides privacy, minimizes interruptions, and allows sufficient time (the time allocated to the appointment is subject to the implications of the alternative diagnosis, if this is known or suspected)
2	Enough time should be allocated for the appointment	
3	Considerations for where the appointment takes place and, where possible, limit opportunities for interruptions, for example, placing telephone on silent/do not disturb and not on a ward	

Most items (40%) reached consensus in Round 1; however, three items (see Table 6, Items 17, 40, and 41) took all three rounds to reach consensus indicating contrasting views from the panelists. Item 17 had 67% and 50% proportion of panelists rate this as “very important” in Round 1 and 2, respectively. Initially, this item proposed: “The doctor should provide a warning to the patient they have something important to discuss.” By Round 2, panelists were reporting their views of this item had changed, for example, a panelist reflected: “Many of these questions, having reflected on them now for a second time, can vary on many different things and it's hard to answer without considering what the scenario is.” Therefore, the wording for this item changed to provide more contextual information, such as “following on from tests completed …” upon which consensus was reached in Round 3. Items 40 and 41 underwent a similar process of review, with item 40 reaching consensus when this was considered something to be addressed at a follow‐up appointment and item 41 when the phrasing was adjusted on the basis of panelist feedback.

**TABLE 6:** Delphi items identified in generic guideline items or multiple sclerosis (MS) misdiagnosis specific.

	Generic guideline item (NICE 2021)	MS misdiagnosis specific item
**I. Preparation**		
1. The doctor should schedule an appointment with the patient as soon as they are aware the patient has been misdiagnosed	✓	
2. The doctor should be familiar with the patient's case by thoroughly reading through their clinical notes prior to the appointment	✓	
3. The doctor should ensure the appointment takes place in an appropriate setting that provides privacy, minimizes interruptions, and allows sufficient time (the time allocated to the appointment is subject to the implications of the alternative diagnosis if this is known or suspected)	✓	
4. The doctor should use an interpreter if they cannot communicate with the patient in the same language	✓	
5. The doctor should ensure the space where the appointment takes place has seating available	✓	
**II. Delivering the information**		
6. The doctor should be unhurried and take time at the start of the appointment to build rapport with the patient	✓	
7. The doctor should actively listen to the patient	✓	
8. The doctor should tailor their information to the unique situation of the patient	✓	
9. The doctor should be honest and transparent in their interaction with the patient. For example, the doctor should say “we do not know your diagnosis” instead of offering an alternative diagnosis if this is the case		✓
10. The doctor should discuss why the diagnosis of MS is in doubt. To do this they might begin by exploring the patient's understanding of their current medical situation. A summary of the reason (e.g., following on from the tests the patient completed) for the appointment and that the new information changes the understanding of the patient's diagnosis should be explained to the patient. In addition, and if this is known, the doctor should provide an explanation of why the diagnosis of multiple sclerosis was initially given to the patient		✓
11. The doctor should use clear and straightforward language when communicating to the patient. For example, avoid using euphemisms such as demyelinating condition/inflammatory disease when referring to multiple sclerosis. The doctor should tailor their communication to the patient and explain the meaning of technical/medical terms to the patient if they need to use these terms in the appointment. The use of analogies may assist the doctor in doing so effectively	✓	
12. The doctor should deliver information in manageable amounts at a time, pausing and checking patient understanding before asking the patient if they are alright to go on	✓	
**III. Managing emotions**		
13. Once the doctor has communicated the news, they should allow the patient time to process this; for example, sitting in silence for a while, or allowing them to express whatever emotion arises		✓
14. If the patient becomes distressed, the doctor should respond with empathy. For example, by eliciting thoughts and feelings in a way that is appropriate to the situation such as saying, “it looks like this is difficult to hear, am I right?”		✓
15. The doctor should provide adequate emotional support for the patient. Where they are unable to do this, they should support the patient to access the appropriate services	✓	
16. The doctor should be prepared to apologise to the patient as most appropriate to the situation. For example, “I'm sorry to be the one to tell you…” or “I can see that this has come as a surprise to you, and I can only imagine how confusing this must be right now. I am sorry that this has not been straightforward for you”	✓	
17. The doctor should acknowledge the impact of removing the diagnosis from the patient might have (especially if the patient has carried the diagnosis for many years)		✓
**IV. Checking understanding and clarifying**		
18. The doctor should ask the patient for their understanding of what they have been told. They could do this by exploring what this news means to the patient, for example, if the patient understands the need to stop treatment, if they are receiving any for MS	✓	
19. The doctor should encourage the patient to ask questions	✓	
**V. Summary, strategy, and signposting**		
20. The doctor should summarize the main points of the appointment for the patient. If known, the reasons for the patient receiving the incorrect diagnosis should be summarized	✓	
21. The doctor should discuss the patient's treatment options given the change in their diagnosis		✓
22. The doctor should clarify if they think a different diagnosis offers a better explanation for the patient's symptoms		✓
23. The doctor should let the patient know if there is not enough information to diagnose their symptoms and discuss whether the patient needs further tests/diagnostic investigation		✓
24. The doctor should consider discussing the implications of removing a diagnosis of Multiple Sclerosis at a follow‐up appointment. For example, legal implications of driving and impact on claiming benefits		✓
25. The doctor could ask what they can do to help the patient adjust to the change in their diagnosis		✓
26. The doctor should let the patient know they will notify the patient's general practitioner (GP) of the change in diagnosis and offer them a copy of the letter.	✓	
27. The doctor should provide the patient with information about what happens next for them, for example, discharge, referral to other services, or a follow‐up appointment with whomever is most appropriate for the patient. This follow‐up appointment may be with the neurologist, a MS nurse, clinical psychologist, or another relevant professional best suited to meet the patient's needs	✓	

## 5 Discussion

Our aim of developing expert consensus‐based guidelines clinicians can use when communicating a misdiagnosis of MS was achieved through a modified Delphi method. Our panel comprised clinicians with a rich experience in the field of MS diagnosis and treatment, and the actual situation of dealing with misdiagnosed cases, alongside published experts in the field of communication, and people with lived experience of receiving a diagnosis of MS. A discussion of the Delphi consensus items follows before the consensus‐based guidelines are presented.

We identified 17 items from the Delphi rounds included in generic guidelines (NICE 2021). This study gained consensus for specific generic guideline items clinicians should consider when preparing for an appointment with a patient who has been misdiagnosed with MS.

We identified 10 items specific to the MS misdiagnosis communication and discuss these in relation to the studies by Coebergh, Wren, and Mumford (2014) and Boissy and Ford (2012), as well as the suggestions available in the breaking bad news literature. Coebergh, Wren, and Mumford (2014) suggestions mirror some of the Delphi items, for example, provide an explanation of the information that has changed the clinician's understanding of the patient's MS diagnosis; explain if the patient requires further investigations or what their treatment pathway consists of; and offering the patient a follow‐up appointment to address any further concerns. Suggestions by Coebergh, Wren, and Mumford (2014), such as inviting the patient to bring a family member to the follow‐up visit, gained consensus as “somewhat important” in the final round for the Delphi panelists and therefore were not included here; however, this may be an appropriate decision to take. This highlights the necessity of clincians using their clinical judgment and knowledge of the patient's situation to inform their practice, rather than using the guidelines prescriptively.

The Boissy and Ford (2012) recommendations place greater emphasis on patients who present with a functional neurological condition and may have been “therapeutically mislabeled” with MS. Two Delphi items overlap with those in Boissy and Ford and are helpful when communicating a misdiagnosis to a patient who likely has a functional diagnosis. The first is to review the medical information collaboratively with the patient, thus providing the clinician an opportunity to be transparent about their clinical reasoning with the patient. Second, discussing the treatment options for the patient, given the change in diagnosis, or, if an alternative diagnosis is unknown, exploring what investigations, including psychological or psychiatric, may help explain the patient's symptoms. It is possible that the patient may have a functional diagnosis comorbid with MS (Walzl, Solomon, and Stone 2022); therefore, the clinician should be aware of this possibility.

There are large methodological variations in the creation of breaking bad news recommendations, as seen in ABCDE (Rabow and Mcphee 1999), SPIKES (Baile et al. 2000), SPIKES‐D (Peixoto, Diniz, and de Oliveira Godeiro 2020), and BREAKS (Narayanan, Bista, and Koshy 2010), whereby items have been collated from literature, based on clinician opinion, or can be found in good practice guidelines. Adapting a current guideline to a particular condition is another way to develop a guideline. For example, recommendations from SPIKES, originally developed for communicating a cancer diagnosis, have been adapted to communicating a dementia diagnosis, SPIKES‐D (Peixoto, Diniz, and de Oliveira Godeiro 2020). Our study included items from all these guidelines in the Delphi Round 1 questionnaire, and panelists chose items that were most appropriate to communicating an MS misdiagnosis. In addition, panelists could suggest changes to the wording, phrasing, and suggest entirely new items.

### 5.1 Recommendations

**FIGURE 1:**
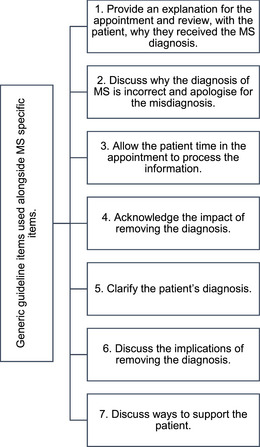
Guidelines for communicating an multiple sclerosis (MS) misdiagnosis.

Consensus‐based guidelines developed from this Delphi study follow a chronological order of the communication process. We recommend that all items be addressed in the MS misdiagnosis communication to ensure systematic and consistent application. Summary of the Delphi recommendations are presented in Figure 1 and further elaborated on below the figure.

 

1. Provide an explanation for the appointment and review with the patient why they received the MS diagnosis. The patient may be anxious about the appointment especially if this is not their usual appointment or they were not expecting the appointment. Informing the patient to the purpose of the appointment orients them to the appointment and what they can expect; the neurologist has information they need to share with the patient. There could be a number of reasons that triggered the review of the diagnosis: A neurologist has retired/left the service, and their cases were reviewed; results from a recent MRI scan (or other test); a clinical audit of cases. Reviewing the patient's diagnosis of MS allows the patient space to be the expert of their experience and the neurologist to acknowledge and validate the patient's symptoms.
2. Discuss why the diagnosis of MS is incorrect and apologize for the misdiagnosis. As well as being a requirement of neurologists’ professional duty of candor, comments from the Delphi feedback round indicated that understanding the reason for the misdiagnosis was crucial in helping patients process the information. A sincere and genuine apology from the neurologist for the distress the misdiagnosis has caused is not an admission of guilt, it is simply the right thing to do. Although this is a statutory requirement and not an admission of guilt, the reservations of the panelists required this item to be placed in both the generic guideline items list and the MS‐ specific recommendations presented here. In highlighting the necessity of apologizing for the misdiagnosis, it is hoped that this becomes embedded in clinical practice, and neurologists are empowered to do so *where appropriate*. Specific wording or phrasing may be obtained through consultation with the service provider's legal department.
3. Allow the patient time in the appointment to process the information. It is possible that the news is completely unexpected and a shock to the patient. It is reasonable to expect the patient to become distressed and consequently to be less effective at processing information than when they are calm. Therefore, give the patient time to process the implications of the information. For the neurologist, sitting in silence and allowing the patient to express whatever emotion comes up for them is likely to be a challenging time in the appointment.
4. Acknowledge the impact of removing the diagnosis. This is important, especially if the patient has carried the misdiagnosis for many years, has been exposed to DMTs, formed an identity around the diagnosis, been active in MS groups, receives financial benefits, or in some other way has significantly been impacted by the diagnosis of MS, which may be quite individual to the patient.
5. Clarify the patient's diagnosis. If the neurologist thinks an alternative diagnosis explains patient's symptoms, then this needs to be discussed with the patient. All necessary information for the treatment pathway should be available to the patient, including further tests, referrals, treatment options, and any other actions. If, an alternative diagnosis is unknown then this, too, should be discussed with the patient including whether the neurologist recommends additional tests or diagnostic investigations.
6. Discuss the implications of removing the diagnosis. Removing a diagnosis is likely to be a shock to the patient, and they are likely to be emotionally overwhelmed. Discussing the practical implications of removing their diagnosis helps the patient to process the implications of the misdiagnosis with the neurologist. The neurologist has the opportunity to support the patient to maintain a realistic and balanced perspective of the adjustments the patient will need to make moving forward. It is also a way for the neurologist to check the patient's understanding of the implications of the misdiagnosis. For example, does the patient understand the need to stop treatment?
7. Discuss ways to support the patient. The neurologist may be required to write letters to insurance companies or other organizations regarding the change in diagnosis. The patient may benefit from counseling to help them adjust to the misdiagnosis. The type of support the patient may need will be unique to them; therefore, this needs to be explored collaboratively. The neurologist has the opportunity to be a source of practical and emotional support to the patient during this significant time.

### 5.2 Strengths and Limitations

Strengths of the study included having equal number of perspectives included. This equity of opinion and perspective was further enhanced by the Delphi methodology of maintaining the anonymity of the panelists from each other. Panelists could share their opinions without being perceived to be judged by other panelists.

Limitations of this study include a lack of data regarding the number of times that neurologists on the panel had personally communicated an incorrect MS diagnosis. The EBE group did not include people misdiagnosed with MS. The authors invite commentary from people who have been misdiagnosed to address the representation of this work.

### 5.3 Future Research

This study addressed a clinical issue of how to communicate an MS misdiagnosis. Future research may update these guidelines for different settings where care for MS patients is provided. This may require the modification of item phrasing or additional items by evaluating the utility of these items for practising clinicians and by patient feedback of their experiences of the communication. Future research may adopt these guidelines for other conditions where misdiagnosis is a known clinical concern. Therefore, future research may develop and refine the recommendations here for the particular field of interest.

## Author Contributions

**Angela Lesley Baufeldt**: Investigation, writing–original draft, methodology, validation, writing–review and editing, software, formal analysis, project administration, data curation, conceptualization, resources. **Nikos Evangelou**: Conceptualization, investigation, writing–original draft, methodology, validation, writing–review and editing, software, formal analysis, project administration, data curation, supervision, resources. **Nima Moghaddam**: Conceptualization, investigation, writing–original draft, methodology, validation, writing–review and editing, software, formal analysis, project administration, data curation, supervision, resources. **Mark Gresswell**: Conceptualization, investigation, writing–original draft, methodology, validation, writing–review and editing, software, formal analysis, project administration, data curation, supervision, resources. **Roshan das Nair**: Conceptualization, investigation, writing–original draft, methodology, validation, writing–review and editing, software, formal analysis, project administration, data curation, supervision, resources.

### Peer Review

The peer review history for this article is available at https://publons.com/publon/10.1002/brb3.70109

## Data Availability

The data that support the findings of this study are available as an e‐Appendix from University of Lincoln. Restrictions apply to the availability of these data, which were used under license for this study. Data are available from the author(s) with the permission of University of Lincoln.

## Search Strategy by Database

### PsycINFO

“Multiple sclerosis” OR (DE “Multiple sclerosis*”)

Diagnosis OR (DE “Diagnosis”) OR “Medical diagnosis” OR (DE “Medical diagnosis”) OR “bad news” OR disclos* OR information* OR “truth disclosure” OR Communicat* OR (DE Communication) OR “Doctor patient communication” OR “Physician patient communication” OR (DE “Information dissemination”) OR (DE “Verbal communication”)

“Therapeutic processes” OR (DE “Therapeutic processes”) OR “Client satisfaction” OR (DE “Client satisfaction”) OR “Patient satisfaction” OR “Quality of care” OR “Quality of services” OR “Client attitudes” OR (DE “Client attitudes”) OR “Patient attitudes” OR “Physician attitudes” OR “Doctor attitudes” OR neurologists OR (DE Neurologists) OR Preference OR (DE Preferences) OR (DE “Healthcare delivery”) OR (DE “Health personnel attitudes”)

### MEDLINE

“Multiple sclerosis” OR (MH “Multiple sclerosis”, “Relapsing‐Remitting”) OR (MH “Multiple sclerosis”) OR (MH “Multiple sclerosis, Chronic Progressive”)

Diagnosis OR “bad news” OR disclos* OR information* OR communicat* OR (MH “Diagnosis”) OR (MH “Health Communication”) OR (MH “Communication”) OR (MH “Nonverbal Communication”)

“therapeutic processes” OR client n2 satisf* OR Patient n2 satisf* OR “Quality of Care” OR “Quality of Services” OR client n2 attitudes OR patient n2 attitudes OR physician n2 attitudes OR doctor n2 attitudes OR preference OR (MH “Patient Satisfaction”) OR (MH “Patient Preference”) OR (MH “Quality of Health Care”) OR (MH “Attitude of Health Personnel”) OR (MH “Physician‐Patient Relations”) OR (MH “Neurologist”) OR “therapeutic process”

## Scopus

“Multiple Sclerosis”

Diagnosis or “Bad News” or Disclos* or Inform* or Communicat*

“therapeutic processes” or (client w/2 satisfaction) or (patient w/2 satisfaction) or “quality of care” or “quality of services” or (client w/2 attitudes) or (patient w/2 attitudes) or (doctor w/2 attitudes) or (physician w/2 attitudes) or (neurologist w/2 attitudes) or “physician‐patient relations” or “doctor–patient relations” or preference

## CINAHL Complete

“Multiple sclerosis” or (MH “Multiple Sclerosis”)

Diagnosis or “bad news” or disclos* or inform* or communicat* or (MH “Diagnosis”) or (MH “Nonverbal Communication”) or (MH “Truth Disclosure”)

“therapeutic processes” or “patient n2 satisfaction” or “client n2 satisfaction” or “quality of care” or “quality of services” or “patient n2 attitudes” or “client n2 attitudes” or “physician n2 attitudes” or “doctor n2 attitudes” or preference or (MH “Patient Satisfaction”) or (MH “Patient Preference”) or (MH “Quality of Health Care”) or (MH “Attitude of Health Personnel”) or (MH “Physician‐Patient Relations”) or (MH “Neurologists”) or “therapeutic process”
